# Unexpected random urinary protein:creatinine ratio results–limitations of the pyrocatechol violet-dye method

**DOI:** 10.1186/1471-2393-13-152

**Published:** 2013-07-17

**Authors:** Dane A De Silva, Anne C Halstead, Anne-Marie Côté, Yasser Sabr, Peter von Dadelszen, Laura A Magee

**Affiliations:** 1 Department of Pathology and Laboratory Medicine, Children’s and Women’s Health Centre of British Columbia and the University of British Columbia, Vancouver, Canada; 2 Department of Obstetrics and Gynaecology, University of British Columbia, Vancouver, Canada; 3 Department of Medicine, Université de Sherbrooke, Sherbrooke, Canada; 4 Department of Specialized Women’s Health, British Columbia Women’s Hospital and Health Centre, 4500 Oak Street, Room 1U59, Vancouver, BC V6H 3N1, Canada; 5 Department of Medicine, University of British Columbia, Vancouver, Canada; 6 Child and Family Research Institute, University of British Columbia, Vancouver, Canada

**Keywords:** Hypertension, Pre-eclampsia, Pregnancy, Protein:creatinine ratio, Proteinuria measurement, Laboratory

## Abstract

**Background:**

For clinicians, it is important to rely on accurate laboratory results for patient care and optimal use of health care resources. We sought to explore our observations that urine protein:creatinine ratios (PrCr) ≥30 mg/mmol are seen not infrequently associated with normal pregnancy outcome.

**Methods:**

Urine samples were collected prospectively from 160 pregnant women attending high-risk maternity clinics at a tertiary care facility. Urinary protein was measured using a pyrocatechol violet assay and urinary creatinine by an enzymatic method on Vitros analysers. Maternal/perinatal outcomes were abstracted from hospital records.

**Results:**

91/233 (39.1%) samples had a PrCr ≥30 mg/mmol, especially when urinary creatinine concentration was <3 mM (94.1%) vs. ≥3 mM (16.4%) (p < 0.001). When using the last sample before delivery, 47/160 (29.4%) had a PrCr ≥30 mg/mmol in diluted urine vs. only 17/160 (15.4%) in more concentrated urine (p < 0.001); PrCr positive results were also more frequent among the 32 (20.0%) women with known normal pregnancy outcome (90.9% vs. 0) (p < 0.001). Using the same analyser, 0.12 g/L urinary protein was ‘detected’ in deionised water. Re-analysis of data from two cohorts revealed substantially less inflation of PrCr in dilute urine using a pyrogallol red assay.

**Conclusions:**

Random urinary PrCr was overestimated in dilute urine when tested using a common pyrocatechol violet dye-based method. This effect was reduced in cohorts when pyrogallol red assays were used. False positive results can impact on diagnosis and patient care. This highlights the need for both clinical and laboratory quality improvement projects and standardization of laboratory protein measurement.

## Background

Accurate proteinuria results are key to the management of adults and children with chronic kidney disease [1], as well as women with a hypertensive disorder of pregnancy [2].

The most commonly used methods of detection of proteinuria in pregnancy are: urinary dipstick by visual or automated testing, random protein to creatinine ratio (PrCr), or 24-hour urine collections. The random PrCr has been recommended as a confirmatory test for pre-eclampsia, defined as a random PrCr ≥30 mg/mmol [2] ( http://www.nice.org.uk/cg107). Since 2009 at our institution, we have used this test more and more for outpatients and inpatients as an easy and timely alternative to 24-hour urine collection for proteinuria determination. However, it has been our clinical impression that there are a number of women who have urinary PrCr values above diagnostic threshold, yet, have normal pregnancy outcomes. We are also aware of possible false positive results outside pregnancy in a community screening study [3].

We sought to investigate our impression that an elevated PrCr result may be associated with normal pregnancy outcome, and if so, to consider potential explanations related to physiology and analytical method.

## Methods

This pragmatic cohort study took place at BC Women’s Hospital & Health Centre in Vancouver, BC from January 27 to March 31, 2011. Consecutive high-risk inpatient or outpatient pregnant women were prospectively evaluated. All women who presented for hypertension in the assessment room or delivery suite or seen at our (primarily morning) ambulatory medicine or high-risk obstetric clinics were included. Women were excluded if they had ruptured membranes or were in labour. As this was a quality improvement study for the hospital laboratory, consent was not required. This study was approved by the University of British Columbia Clinical Research Ethics Board (H10-02691).

Random midstream urine samples obtained as part of normal clinical care were split into two aliquots; women are not routinely asked to provide first-voided urines, but rather, they provide the sample when they arrive at the clinic. The first was used for urinary dipstick testing. The second aliquot was sent to the hospital laboratory where it was centrifuged at a speed of 1500 rpm for 5 minutes, and then tested for urinary PrCr in batches on an automated analyser (Vitros 5,1 FS or Vitros 5600, Ortho-Clinical Diagnostics, Rochester, NY) as is standard laboratory procedure. Testing included urinary creatinine (using an enzymatic method) and protein (using a pyrocatechol violet molybdate dye-binding method), followed by calculation of the urinary PrCr [4,5]. The manufacturer lists limits of detection of 0.05 g/L for protein and 0.106 mmol/L for creatinine. The coefficients of variation of the protein and creatinine assays are 2.9% at a concentration of 0.3 g/L and 2.0% at a concentration of 5.3 mM, respectively. In addition to the routinely collected, visually interpreted urinary dipstick testing performed in the clinic, clinicians also received the results of the urine PrCr.

Hospital records were used to abstract maternal and perinatal outcomes. Maternal outcomes included demographics, parity, multiple pregnancy, details of the indication for attendance at the high-risk clinic and other medical co-morbidities, antenatal complications such as haemorrhage or preterm labour, and delivery information including mode of delivery. Perinatal outcomes included stillbirth, neonatal death, birthweight, and neonatal intensive care unit (NICU) admission.

In our exploratory analysis, we plotted the creatinine concentration (mM) against the PrCr (mg/mmol) for all random urine samples submitted (as some women provided more than one urine sample). Then we focused on using the last random urine sample submitted before delivery. In response to the results, we performed sensitivity analyses in which we included only urine samples with a specific gravity (SG) >1.010 and samples from women with no dipstick hematuria. Samples from women with normal pregnancy outcome (i.e., no evidence of pre-eclampsia and a term delivery of an appropriately grown infant) were highlighted in all analyses. We then performed a dilution study on a new random urine sample with a protein result in the low-mid area of the Vitros assay analytical range of 0.05 – 2.00 g/L. 1:2, 1:3, 1:4, and 1:5 dilutions of the urine were made using either deionised water or saline as the diluent and tested for urinary protein on the analyser. To verify whether our findings were related to the urinary protein method used, we repeated our statistical analyses in two separate published cohorts of women in which proteinuria was determined using a pyrogallol-based dye-binding urine protein assay [6,7]. The Pearson’s Chi-squared test and Fisher’s exact test were used to calculate p values where appropriate.

## Results

The 160 women in the study cohort provided 233 samples at one/more antenatal visits, although most women (114 or 71.3%) provided only one sample. Table 1 presents the baseline characteristics of this study cohort. Most women had singleton pregnancies, were evaluated as outpatients in the second trimester, and were not receiving antihypertensive therapy at the time of urine sampling. One third of women had a hypertensive disorder of pregnancy at sampling, most commonly pre-existing hypertension.

**Table 1:** Baseline characteristics of the 160 women in the study cohort (N (%) or median [interquartile range], as appropriate)

	**N = 160 women**
**Maternal characteristics**	
Maternal age (yr)	34 [31–37]
Primiparous	75 (46.9%)
Multiple pregnancy	5 (3.1%)
**Pregnancy characteristics at the time of urine sampling**	
Outpatient	131 (81.9%)
N samples collected before 1300 hours	128 (80%)
Gestational age when urine sample taken	24 [20–32]
On antihypertensive therapy	31 (19.4%)
Hypertensive disorder at sampling	61 (38.1%)
* Pre*-*existing hypertension only*	25 (15.6%)
* Pre*-*existing hypertension with baseline proteinuria*	3 (1.9%)
* Gestational hypertension without dipstick proteinuria*	19 (11.9%)
* Pre*-*eclampsia* (*including HELLP syndrome*)	14 (8.8%)
Medical co-morbidities other than hypertension	
* Women with one*/*more co*-*morbidity*	60 (37.5%)
* Women with two or more co*-*morbidities*	21 (13.1%)
Specific co-morbidities (N women)	
* Diabetes* (*pre*-*gestational or gestational*)	16 (10%)
* Pre*-existing kidney disease*	8 (5%)
* Other co*-*morbidities*†	47 (29.4%)
**Pregnancy outcome after urine sampling (N = 112 women with known pregnancy outcome)**	
Delivery at BCWH or post-partum follow-up	112 (70%)
Not known or lost to follow-up	48 (30%)
Miscarriage or elective termination	4 (3.6%)
Stillbirth	1 (0.9%)
Placental abruption or other APH	3 (2.8%)
Preterm pre-labour rupture of membranes	4 (3.7%)
Chorioamnionitis	1 (0.9%)
Gestational age at delivery (wk)	38 [37–39]
* Delivery at* <*37 wk*	17 (15.7%)
Caesarean section	52 (48.1%)
Small for gestational age infants	19 (17.6%)
Neonatal intensive care unit admission	12 (11.1%)

*APH* (antepartum haemorrhage), *BCWH* (British Columbia Women’s Hospital and Health Centre), *HELLP* (haemolysis, elevated liver enzyme, low platelet) syndrome.

* No women had acute kidney injury. All eight women with chronic kidney disease were outpatients. Their serum creatinines ranged from 48-207 μM with only one woman having a pre-recruitment serum creatinine >90 μM.

† Other medical co-morbidities included the following (N women): thyroid disorders (15), systemic lupus erythematosus (6) or another connective issue disorder (4), depression (5), anemia (4), antiphospholipid antibody syndrome (3), polycystic ovarian syndrome (2), multiple sclerosis (2), immune thrombocytopenia (2), Raynaud’s (2), diabetes insipidus (1), hypercholesterolemia (1), biliary colic (1), deep vein thrombosis (1), polycythemia vera (1), polymyositis (1), scleroderma (1), pulmonary fibrosis (1), Addison’s disease (1), Crohn’s disease (1), celiac disease (1), histiocytosis (1), hepatitis B (1), hyperaldosteronism (1), asymptomatic bacteriuria (1), and/or solitary kidney (1).

### Preliminary analysis for all samples

**Figure 1:**
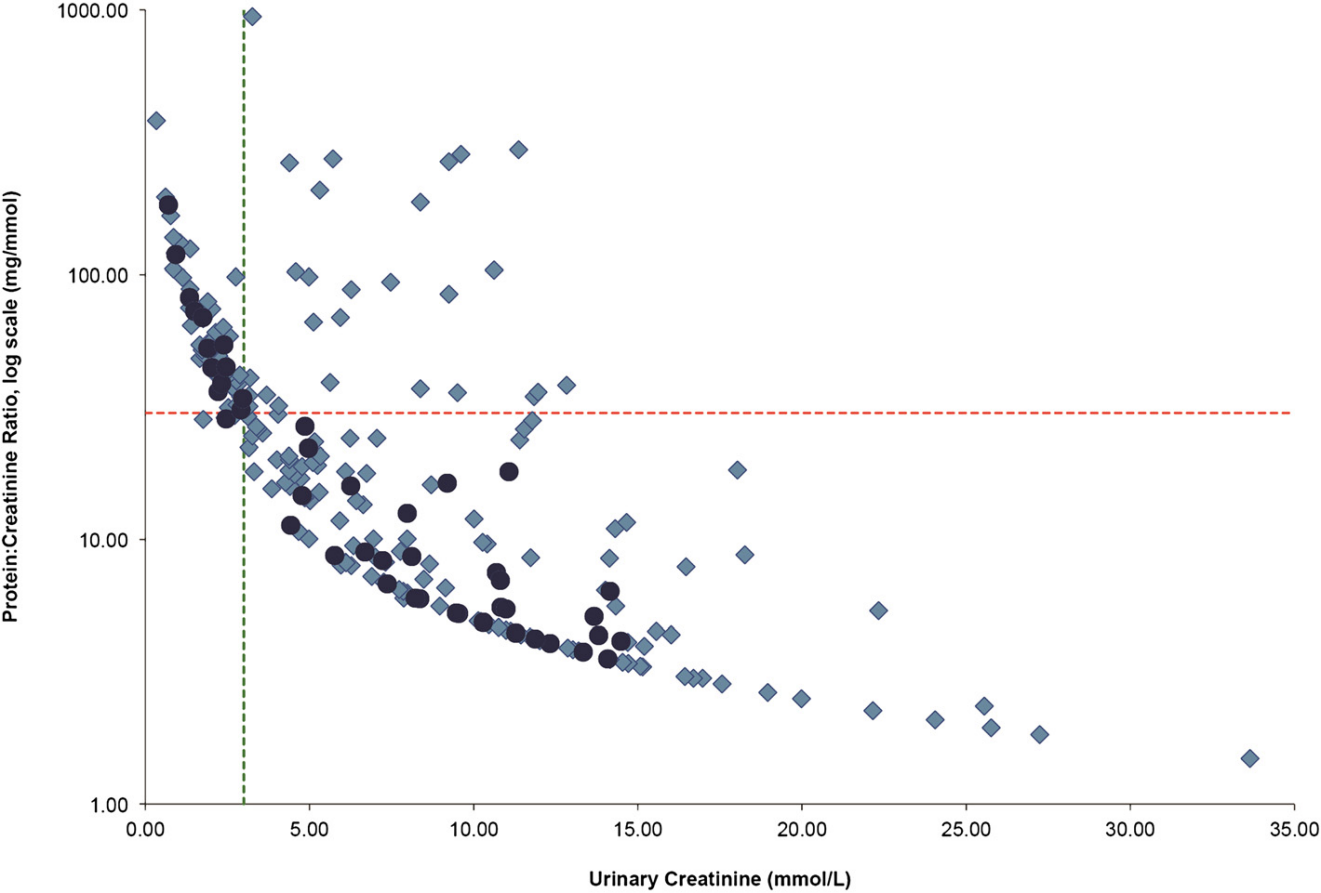
**Random urinary protein**:**creatinine ratio (PrCr, mg/mmol) according to urinary creatinine concentration for all random urine samples (N = 233) (mmol/L).** The horizontal dotted line represents a PrCr of 30 mg/mmol, the current cut-off for detection of 0.3 g/d of proteinuria. The vertical dotted line represents a urinary creatinine concentration of 3 mmol/L. Women with known normal pregnancy outcome are represented by the darker circles.

All 233 random urine samples were assayed for PrCr of which 91 (39.1%) were ≥30 mg/mmol. 50 (21.5%) samples had urine protein below the assay detection limit, and their PrCr values were calculated using the assay cut-off for protein of 0.05 g/L; all 50 had PrCr <30 mg/mmol. The relationship between urinary creatinine concentration and the random PrCr result is presented for all samples in Figure 1. Low urinary creatinine concentration (defined as those <3 mM) was associated with higher urinary PrCr results (≥30 mg/mmol) regardless of pregnancy outcome.

### Detailed analysis for last sample before delivery

**Figure 2:**
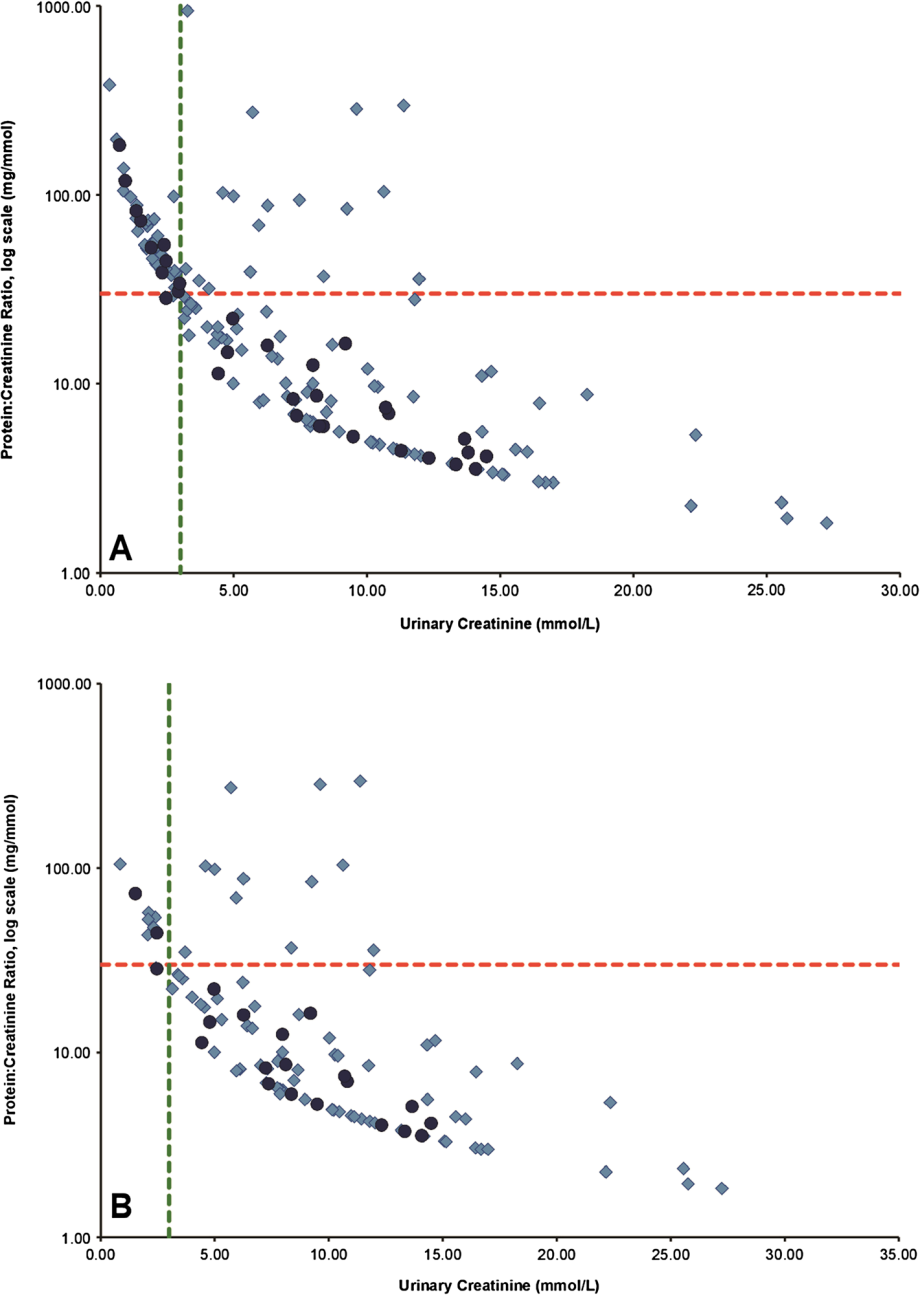
**Random urinary protein:creatinine ratio (PrCr, mg/mmol) according to urinary creatinine concentration using the last sample before delivery (mmol/L). A)** Last urine samples provided. **B)** Excluding urine samples with specific gravity (SG) ≤ 1.010. The horizontal dotted line represents a PrCr of 30 mg/mmol, the current cut-off for detection of 0.3 g/d of proteinuria. The vertical dotted line represents a urinary creatinine concentration of 3 mmol/L. Women with known normal pregnancy outcome are represented by the darker circles.

The relationship between urinary creatinine concentration and the random PrCr is presented for the last sample provided before delivery in Figure 2. 50 (31.3%) samples were dilute (i.e., with urine creatinine concentration <3 mM) and 55/159 (34.6%) samples had dipstick specific gravity (SG) ≤1.010. Low urinary creatinine concentration was still associated with higher urinary PrCr results (Figure 2A). The same relationship was seen for samples with urinary dipstick SG >1.010 (Figure 2B), or when samples with dipstick hematuria and leukocytes were excluded (Figure available upon request).

**Figure 3:**
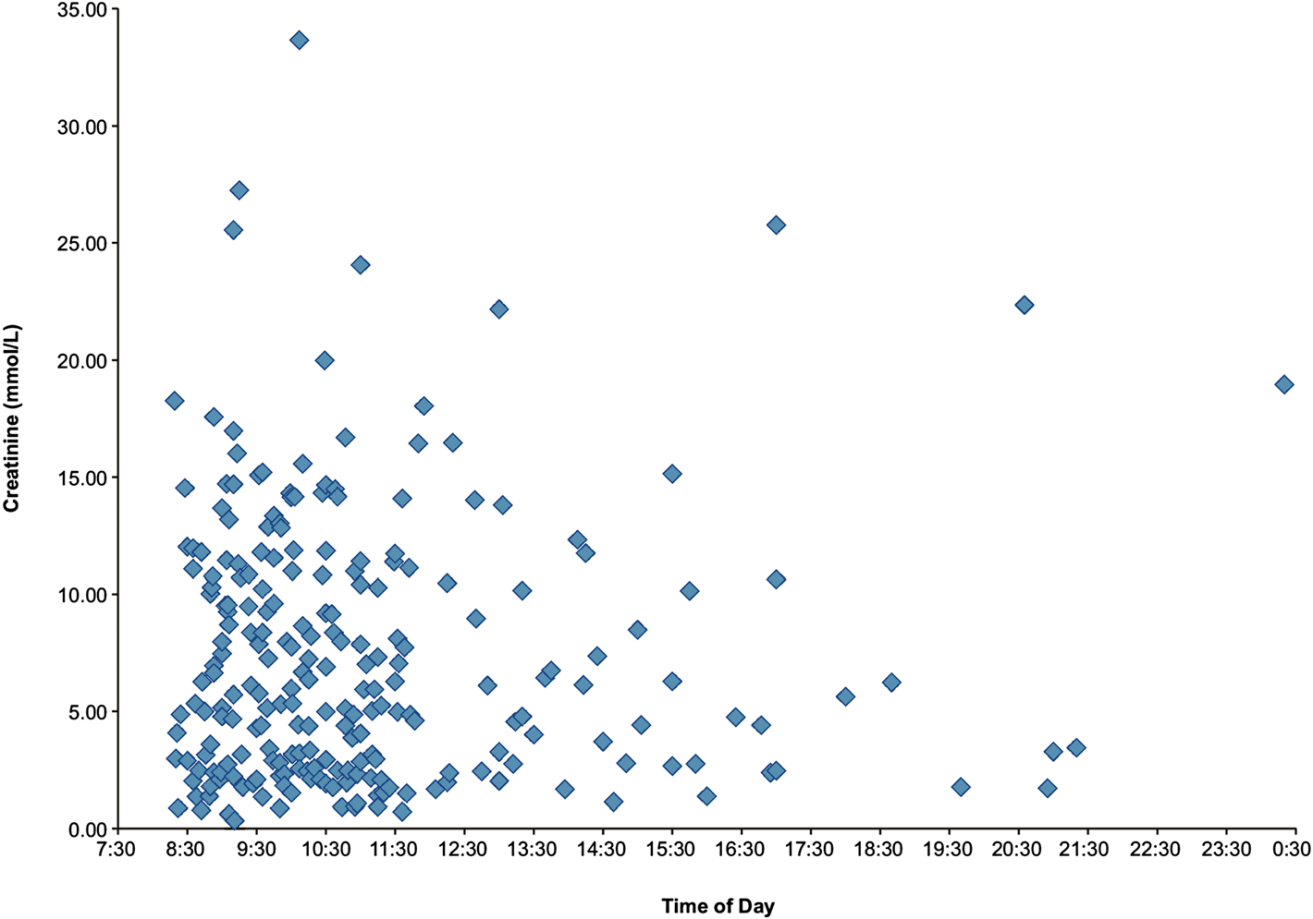
Urinary creatinine concentration (mmol/L) according to the time of day of urine sampling (24 hour clock).

In response to the relationship to urine concentration we found, we looked at the time of day urine samples were collected. Most women (128/160; 80.0%) provided random urine samples before 1300 hours, as the outpatient clinics were run in the morning. The random urine samples collected in the morning were not more concentrated in the morning when looking at urinary creatinine concentration according to time of the collection during the day (Figure 3).

There were no clinical concerns about urinary tract infection but women could have had renal disease. By automated dipstick, 21/159 samples tested (13.2%) were positive for red blood cells (RBC); urine microscopy was routinely performed for the 29 inpatients only if samples tested positive (as per standard laboratory practice) and 2/16 (12.5%) samples had >3 RBC per high power field. 53/159 samples (33.3%) tested positive for leukocyte esterase, which tests for presence of white blood cells. Of those that had urine microscopy performed, 7/16 (43.8%) samples had >5 WBC per high power field. Only one (0.6%) tested positive for nitrites. No sample had a positive urine culture for bacteria.

Table 2 presents the number of samples with PrCr ≥ 30 mg/mmol as a percentage of those with low (<3 mM) or high (≥3 mM) urine creatinine concentration, for the groups presented in Figures 1 and 2A-B. Also presented are the relationships between urinary creatinine concentrations and PrCr in two other cohorts of women who had been recruited from our centre for different studies.

**Table 2:** N (%) of samples with urinary PrCr ≥ 30 mg/mmol according to urinary creatinine concentrations, in current study and two other study cohorts from the same (our) institution

	**N urine samples**	**Urinary creatinine concentration N (%) samples**		
		**<3 mM**	**≥3 mM**	**p value**
**Current study** (N = 160 women)				
All samples in current study	233	64/68 (94.1%)	27/165 (16.4%)	<0.001
All samples from women with known normal pregnancy outcome	45	13/14 (92.9%)	0/31	<0.001
Last urine sample from all women	160	47/50 (94.0%)	17/110 (15.4%)	<0.001
Last urine sample from women with known normal pregnancy outcome	32	10/11 (90.9%)	0/21	<0.001
Last urine sample from women with urinary SG >1.010	104	9/11 (81.8%)	12/93 (12.9%)	<0.001
Last urine sample excluding samples with dipstick hematuria or leukocytes	98	37/39 (94.9%)	10/59 (16.9%)	<0.001
**24 hour completeness cohort**[6] (N = 198 women)				
Last sample per woman	197	12/18 (66.7%)	99/179 (55.3%)	0.354
**PIERS study cohort**[7] (N = 931 women)				
**Spot PrCr** (all samples)		N = 601	N = 1285	
Pyrogallol red proteinuria assay	2432	482/655 (73.6%)	1089/1777 (61.3%)	<0.001
Vitros proteinuria assay	468	119/122 (97.5%)	196/346 (56.6%)	<0.001
**24**-**hour PrCr** (all samples)		N = 103	N = 331	
Pyrogallol red proteinuria assay	607	92/125 (73.6%)	313/482 (64.9%)	0.067
Vitros proteinuria assay	43	11/11 (100%)	18/32 (56.3%)	0.008

PrCr (protein:creatinine) ratio.

### 24 hr urinary completeness cohort

**Figure 4:**
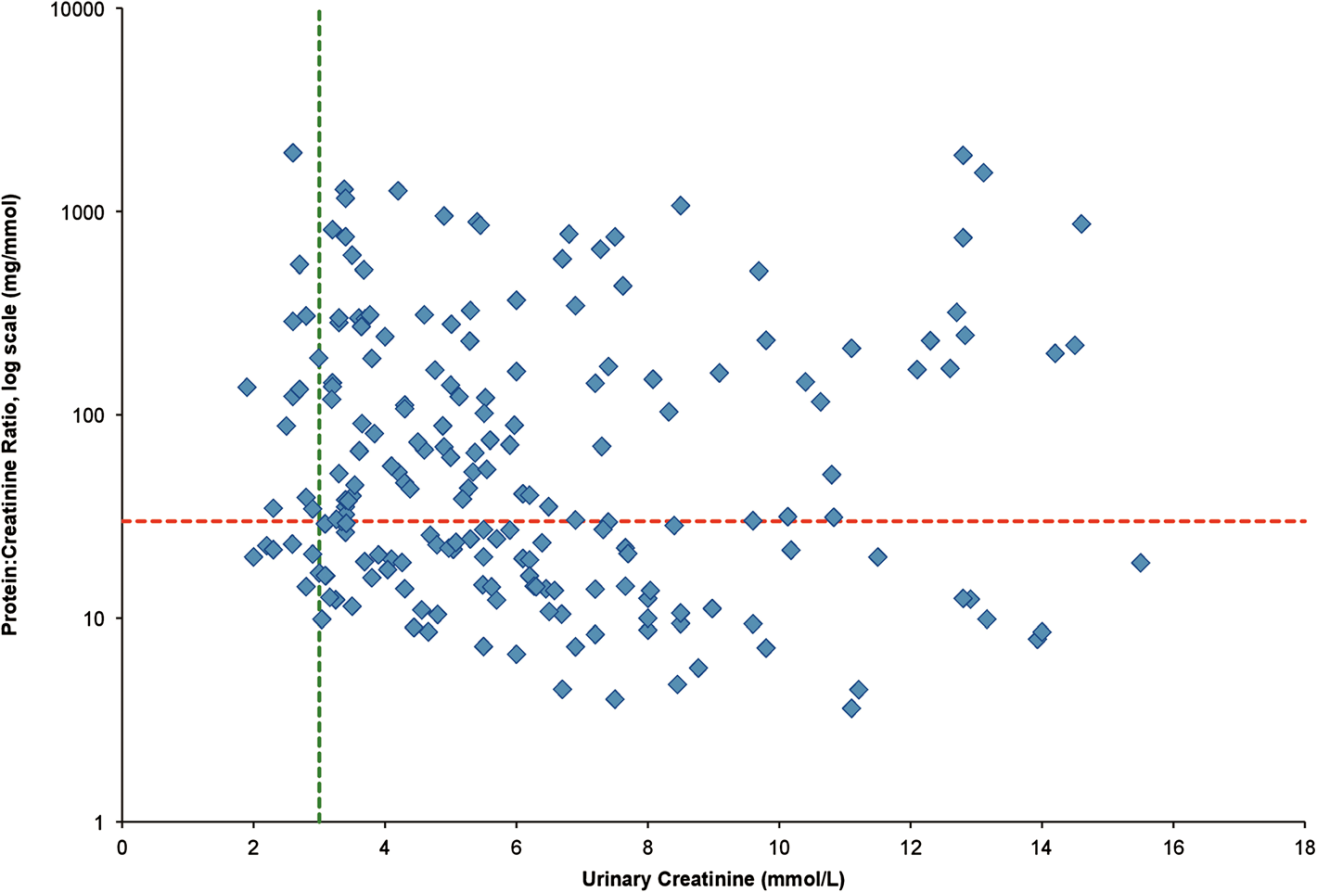
**All 24**-**hour PrCr results using the pyrogallol red dye from a published cohort of women with 24-hour urine collection**^**6**^**.** The horizontal dotted line represents a PrCr of 30 mg/mmol, the current cut-off for detection of 0.3 g/d of proteinuria. The vertical dotted line represents a urinary creatinine concentration of 3 mmol/L.

As our hospital used a manual pyrogallol red dye-based urine protein assay until 2009, we explored the relationship between urinary creatinine concentrations and (24-hour) PrCr in a cohort of 198 women who had a 24-hour urine collection for protein at our hospital between 1997 and 2004 [6]. This cohort consisted of pregnant women with a hypertensive disorder (including 63.1% with pre-eclampsia and 23.7% with pre-existing hypertension). The creatinine assay was similar (Vitros 250/950, Ortho Clinical Diagnostics, Rochester, NY.) 161 (81.3%) of women were inpatients, and 24-hour urine creatinine concentration was <3 mM in 17 (10.6%). There was no clear pattern of increased urine PrCr at low urine creatinine concentration as shown in Table 2 and presented graphically in Figure 4.

### PIERS cohort

In a dataset of women admitted to hospital with pre-eclampsia or who developed pre-eclampsia and had not yet presented with one of the serious outcomes [7], the inflation of urinary PrCr (measured on 24-hour urine samples) at low urinary creatinine concentration was marked for samples run using the Vitros method (N = 468 samples), but the effect largely disappeared for those run before 2009 using the pyrogallol red method (N = 2432 samples) (Table 2). These results are available online (Additional file 1: Figure S1). For random PrCr results, the same pattern of inflated PrCr values was seen at low urinary creatinine concentration with Vitros but to a less extent with pyrogallol red (Table 2, Additional file 2: Figure S2).

To investigate the role of the Vitros protein assay on our results, dilution studies were undertaken on a new random urine sample using deionised water as the diluent (as recommended by the manufacturer). There was an excellent correlation between expected and measured concentrations of protein (R^2^ = 0.99927). However, as shown in Table 3, diluted urine consistently showed higher protein concentrations than expected, as reflected by the ‘recoveries’ (measured protein result as a percentage of calculated expected concentration). The problem was less apparent when the diluent used was saline, which has a higher ionic strength than water. The analyser gave protein results when pure deionised water and saline were tested, at concentrations of 0.12 g/L and 0.06 g/L, respectively.

**Table 3:** **Dilution studies using a standard urine specimen containing 0**.**335 g**/**L of protein and either deionized water or saline as the diluent**

		**Measured proteinuria concentration**			
**Dilutions of urine sample**	**Expected proteinuria concentrations**	Water as the diluent*†	**Recovery of proteinuria as a % of expected**	**Saline as a diluent‡**	**Recovery of proteinuria as a % of expected**
Undiluted	0.335 g/L	n/a	n/a	n/a	n/a
1:2	0.168 g/L	0.242 g/L	144%	0.183 g/L	109%
1:3	0.112 g/L	0.199 g/L	178%	0.140 g/L	125%
1:4	0.084 g/L	0.183 g/L	219%	0.118 g/L	141%
1:5	0.067 g/L	0.172 g/L	257%	0.106 g/L	158%

n/a (not applicable).

* Water measured on its own yielded a protein concentration of 0.12 g/L.

† The unexpectedly high result for underlines the problem with dilute samples. The assay is optimized for measuring urine, not aqueous samples. Method calibrators contain protein (bovine serum albumin) added to a synthetic urine matrix with inorganic salts, a polymer, preservatives and stabilizers. Protein containing calibrators are targeted at 0.80 and 2.10 g/L and the curve is extrapolated down. The low end is verified with a calibrator at less than 0.15 g/L. In the Vitros methods, the lower analytical limit is defined by performance (precision and linearity studies) at a low level in urine or synthetic urine samples. The dye works optimally at the ionic strength of non-dilute urine (due to the various ions present), so that protein as low as 0.05 g/L can be detected with confidence. But in a low ionic strength solution (such as pure water or saline), the dye binding characteristics result in false detection of protein.

‡ Saline on its own yielded a proteinuria concentration of 0.06 g/L.

## Discussion

In our pragmatic study of primarily outpatient women attending high-risk pregnancy clinics, we found that the random urinary PrCr was inflated when urinary creatinine concentration was <3 mM, regardless of pregnancy outcome. This relationship was indeed surprising, as taking a ratio of protein and creatinine concentrations should correct for urinary dilution.

In seeking an explanation for increased PrCr results at low urinary creatinine concentrations, we considered both physiological and analytical explanations. First, we saw inflated random urinary PrCr results when analyses were restricted to women with urine samples with SG >1.010 or those with no dipstick hematuria or leukocytes. Second, considering that proteinuria in pregnancy is measured primarily to detect pre-eclampsia, either *de novo* or superimposed on pre-existing hypertension (present in 15% of our cohort), it must be noted that women with pre-eclampsia tend to be intravascularly volume *contracted* so if anything, they should have decreased urine volumes (or even oliguria) and *elevated* (not decreased) urinary creatinine concentrations. Many of these women are placed on bedrest, but this would not account for decreased urinary creatinine concentration because the natural history of pre-eclampsia is too short to have a significant impact on muscle mass (and urinary creatinine excretion); women at term gestations are delivered right away [8] and those remote from term are delivered for maternal/fetal reasons either right away (40%) or on average, within 10–14 days (30%) [9]. Third, in considering the role of analytical methods, in two other patient cohorts from our centre (which used pyrogallol red dye-based protein assays until 2009, and Vitros thereafter) random urinary PrCr results were inflated in samples tested by the Vitros compared to those tested using pyrogallol red. Of particular note, 10% of 24-hour urine samples were dilute [6] and thus, subject to the same problem as the random urine samples in the current study. Finally, dilution studies showed that using our pyrocatechol violet molybdate dye-binding method on the Vitros analyser, there was overestimation of urine protein as dilution increased, and even pure water and saline contained ‘measurable’ protein. This led us to the conclusion that our random urinary PrCr results were inflated in dilute urines because of falsely high urine protein results.

These results helped to explain both our clinical observations that some women with elevated random urinary PrCr results had normal pregnancy outcome, as well as the findings of a previous report of unexplained proteinuria of 0.38 g/d associated with high water intake (and low 24-hour urinary creatinine concentration of 2.9 mM) in 63 people in a community screening study [3]. In that published study, the Vitros 950 autoanalyser was used to measure protein, and when 56 of the subjects agreed to decrease their water intake, urine protein significantly decreased (to 0.16 g/d, associated with an increase in urinary creatinine concentration to 6.9 mM).

The manufacturer of the Vitros urine protein assay (Ortho-Clinical Diagnostics, Rochester, NY) has documented that when urinary SG is ≤1.010, measurement of 24-hour urine protein excretion may be falsely elevated as a limitation of the assay [5]. We found, however, that urine creatinine <3 mM was more effective than urine SG ≤1.010 for identifying dilute samples in which random urinary PrCr was overestimated; the inaccuracy of urine dipstick SG compared to other measures of urine concentration is well documented [10], whereas the use of creatinine to correct for variability in urine concentration is the premise of PrCr assessment [1]. The overestimation is attributed to the fact that the accuracy of pyrocatechol violet molybdate dye-binding relies on the ionic strength of the urine. Lower ionic strength (as seen in dilute urine) may result in more dye-binding and higher measured protein concentrations [11]. This overestimation by Vitros of protein concentration in dilute urine has been previously published [11,12]. Consistent with the manufacturer’s explanation was our observation that when saline was used as the diluent in our dilution studies (thus, offering some ionic strength from the sodium chloride), inflation of urinary protein measurement seen with use of deionised water was attenuated. Although this study showed that proteinuria results were inflated in dilute urine because of analytical bias, it is to be noted that the use of creatinine may also fail to correct for urinary dilution in very dilute urines for other analytes, for example, as demonstrated for urinary albumin [13].

Our 2008 review of the diagnostic accuracy of the random urinary PrCr concluded that it is a “reasonable” rule-out test for detecting proteinuria of ≥0.3 g/d in hypertensive pregnancy [14]. Our findings in the current study should have no impact on this conclusion. Although that review could not examine the impact of urine protein assay method on the results because of incomplete reporting of methods, none of the included studies used the current Vitros method; five used the dye pyrogallol red.

It should be highlighted that this is not a problem restricted to random urinary samples; 10% of 24-hour urine collections may be dilute (i.e., have urinary creatinine <3 mM) [6] and proteinuria is assayed in 24-hour urine samples by taking a sample, measuring the urine protein concentration, and then multiplying it by the volume of urine submitted to get the value for protein excretion in g/d.

The impact on maternal management of a false positive proteinuria result may include patient anxiety and greater use of health care resources due to enhanced maternal and fetal surveillance (as a diagnosis of pre-eclampsia is associated with both maternal and perinatal risk), hospital admission, and/or earlier delivery [2]. Higher costs related to misclassification of renal function related to creatinine standardization have also been previously published [15].

The first strength of our study is that in our current study cohort, we maximised generalisability by assessing a broad spectrum of (primarily outpatient) high-risk patients with and without significant proteinuria as diagnosed by a random urinary PrCr of ≥30 mg/mmol. We also expanded our analyses to two other cohorts of primarily inpatient women with pre-eclampsia. Second, we performed an additional exploratory dilution study using the Vitros proteinuria method, and validated our result in two separate cohorts of women with proteinuria measurement by a different dye-based method (i.e., pyrogallol red).

Some would point out that the random urinary PrCr result was not compared to 24-hour urinary protein; however, it must be acknowledged that the 24-hour urine collection is frequently incomplete in pregnancy and as such, is no longer considered to be the gold standard for diagnosis of proteinuria in pregnancy [6].

## Conclusions

In conclusion, our Vitros random urinary PrCr results were inflated in dilute urines because of overestimation of urine protein as a result of technical limitations of the pyrocatechol violet dye-based assay, as confirmed by dilution studies. We observed this phenomenon in another cohort in which Vitros was used. However, the effect was greatly reduced when examining data from two other independent cohorts from our centre in which pyrogallol red was the dye used in the urine protein assay.

### Future directions

Our study highlights the need for both follow-up of clinical observations as well as collaboration between clinicians and laboratory medicine specialists. At our tertiary perinatal unit where proteinuria assessment is done largely to detect proteinuria in pregnancy, we will be reassessing our assay. Pending future clarification, it may be prudent for clinicians to focus on first voided urines when at all possible, and when not, to consider the potential for falsely elevated random urinary PrCr results when urinary creatinine concentration is <3 mM. This cautionary note also applies to 24-hour urine collections. It is not known whether urinary dilution has an impact on the accuracy of proteinuria assessment by other methods (dye-based or other), for both random urinary PrCr testing and 24-hour urinary protein determination. Given the importance of proteinuria assessment both in and outside pregnancy, laboratory standardization should be vigorously pursued.

## Competing interest

The authors declare that they have no competing interest.

## Authors’ contributions

LAM conceived the idea for the project. LAM, ACH, and DAD were involved in planning, while DAD carried out and performed the experiments. DAD and YS collected data, and DAD performed the analyses. LAM, AMC, and DAD were responsible for manuscript preparation, with all authors (DAD, ACH, AMC, YS, PvD, LAM) contributing to the interpretation of the data and manuscript preparation. All authors read and approved the final manuscript.

## Pre-publication history

The pre-publication history for this paper can be accessed here:

http://www.biomedcentral.com/1471-2393/13/152/prepub

## Supplementary Material

Additional file 1: Figure S1 Random urinary protein:creatinine ratio (PrCr, mg/mmol) according to urinary creatinine concentration (mmol/L), presented by type of urine protein assay. **A)** Pyrogallol red urine assay. **B)** Vitros urine protein assay. The horizontal dotted line represents a PrCr of 30 mg/mmol, the current cut-off for detection of 0.3 g/d of proteinuria. The vertical dotted line represents a urinary creatinine concentration of 3 mmol/L. Click here for file

Additional file 2: Figure S2 Protein concentration result (g/L) using a standard urine specimen containing 0.335 g/L of protein according to various sample:diluent ratios of either deionized water or saline as the diluent (Table 3). Click here for file
