# Supplementary figures and images for: Unexpected random urinary protein:creatinine ratio results–limitations of the pyrocatechol violet-dye method
Source: BMC Pregnancy Childbirth. 2013 Jul 17;13:152. doi: 10.1186/1471-2393-13-152 (PMC3733961; doi:10.1186/1471-2393-13-152)

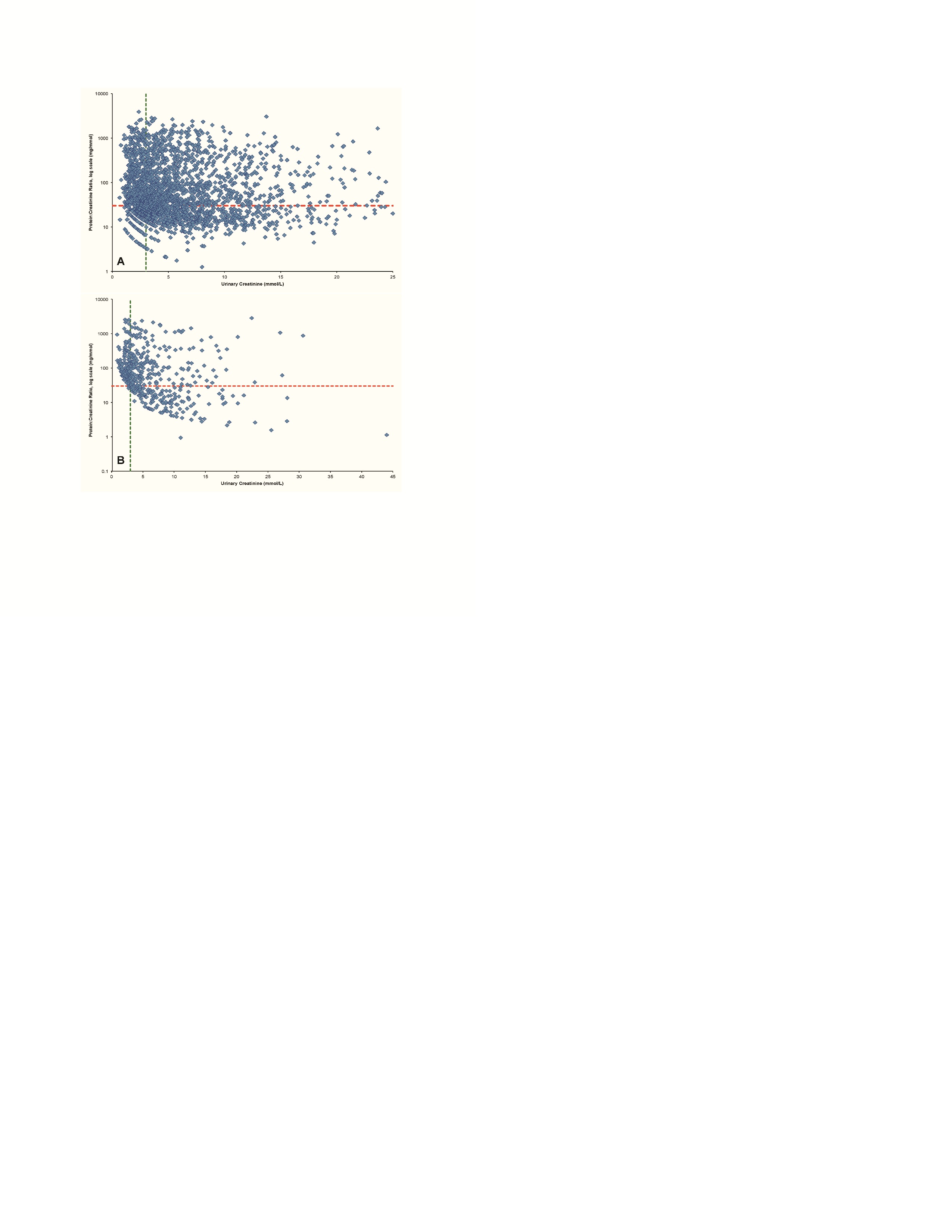

Supplement: Additional file 1: Figure S1 — Random urinary protein:creatinine ratio (PrCr, mg/mmol) according to urinary creatinine concentration (mmol/L), presented by type of urine protein assay. A) Pyrogallol red urine assay. B) Vitros urine protein assay. The horizontal dotted line represents a PrCr of 30 mg/mmol, the current cut-off for detection of 0.3 g/d of proteinuria. The vertical dotted line represents a urinary creatinine concentration of 3 mmol/L. [file 1471-2393-13-152-S1.jpeg]

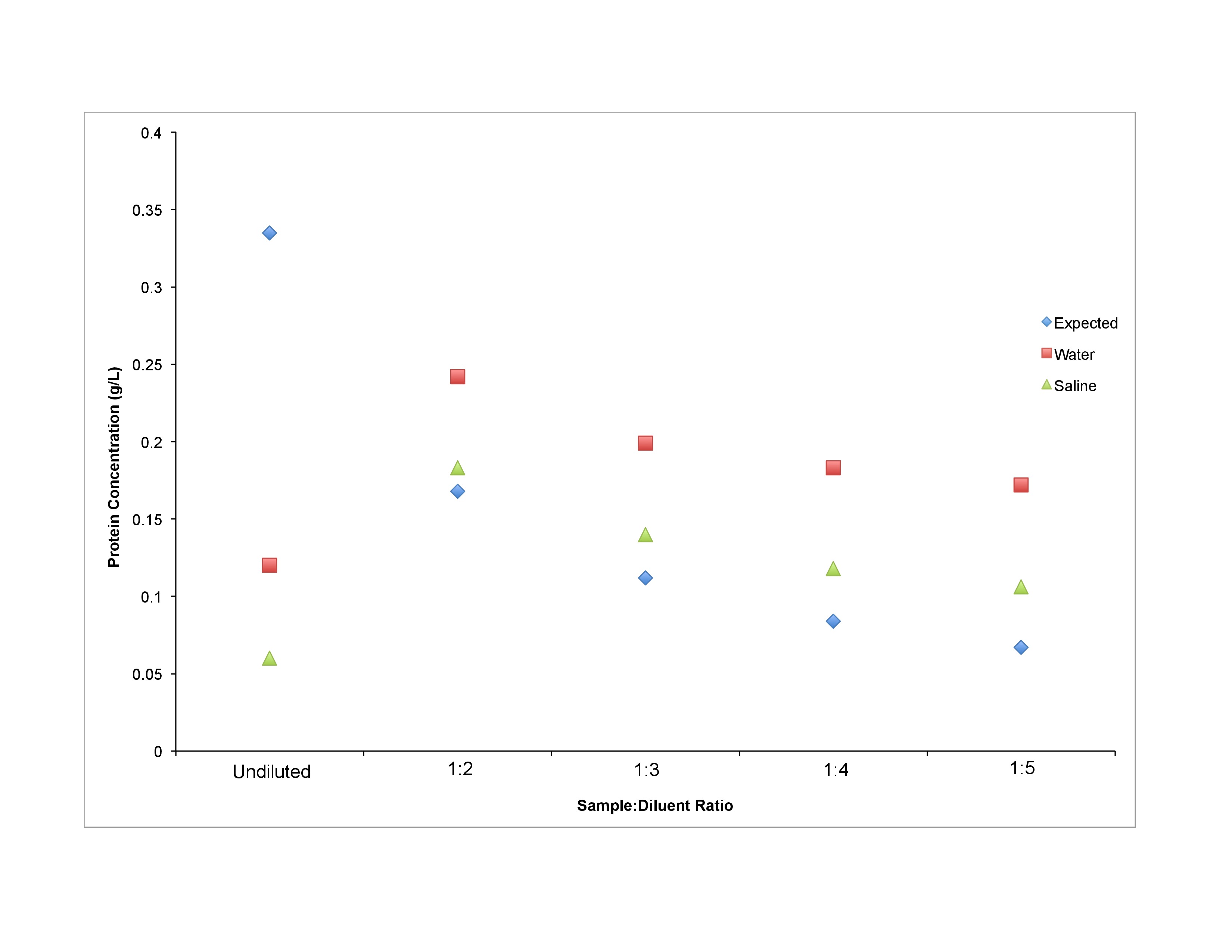

Supplement: Additional file 2: Figure S2 — Protein concentration result (g/L) using a standard urine specimen containing 0.335 g/L of protein according to various sample:diluent ratios of either deionized water or saline as the diluent (Table 3). [file 1471-2393-13-152-S2.jpeg]
